# Effects of a SWELE program for improving mental wellbeing in children and adolescents with special educational needs: protocol of a quasi-experimental study

**DOI:** 10.1186/s12887-024-05288-8

**Published:** 2024-12-06

**Authors:** Regina Lai Tong Lee, Sally Wai Chi Chan, Yuen Yu Chong, Steven Wai Ho Chau, Kai Chow Choi, Wai Tong Chien

**Affiliations:** 1 The Nethersole School of Nursing, Faculty of Medicine, The Chinese University of Hong Kong, Hong Kong, China; 2 School of Nursing and Midwifery, College of Health, University of Newcastle, Medicine & Wellbeing, Callaghan, NSW Australia; 3 Tung Wah College, Hong Kong, China; 4 Department of Psychiatry, Faculty of Medicine, The Chinese University of Hong Kong, Hong Kong, China

**Keywords:** Unstructured play, Mindfulness-based interventions, Children and adolescents, Special educational needs, Mood, Anxiety, Social skill, Playfulness

## Abstract

**Background:**

Unstructured play has also been found effective on promoting positive emotions and emotional competence among children and adolescents with special educational needs (SEN). Unstructured Outdoor Play coupled with Mindfulness-based Interventions via ‘Supporting Wellness in E-Child Learning Environments’ (SWELE) program can foster the mental health and wellbeing of children and adolescents with SEN. Currently there is a lack of the unstructured play with mindfulness-based interventions in the special schools in Hong Kong. Thus, aim of the SWELE program is to examine the effects of unstructured play with mindfulness-based interventions—(SWELE) program combining unstructured outdoor play activities with mindfulness-based interventions to promote mental wellbeing among SEN children and adolescents.

**Methods:**

This 16-week SWELE program focuses on combining unstructured outdoor play activities with mindfulness-based interventions to promote mental wellbeing among children and adolescents with SEN. Training workshops for schoolteachers, school principals, parents and student mental health ambassadors in each special school together with a 16-week SWELE program and each last 45–60 min. Unstructured play allows students the freedom to explore, create and discover without predetermined rules or guidelines. Outdoor unstructured play activities include naturally run, jump and move on a playground, riding a bike, climbing trees, playing tag, playing with blocks, colour, water play, and boxes. A convergent parallel mixed-methods design both with a quantitative measure using a single group pre- and post-tests quasi-experiment behavioural observational method to preliminarily evaluate the impact of SWELE Program on SEN children’s and adolescents’ mental wellbeing; and with a qualitative design to conduct eight focus group discussions including schoolteachers, school principals, parents, SEN students, school nurses from six selected special groups and to explore their perceptions and experiences after participated the SWELE program.

**Discussion:**

The purpose of this protocol is to examine the effects of a 16-week SWELE program on the mood, anxiety level, social skills and playfulness behaviours among children and adolescents with SEN in the special schools in Hong Kong. From the evaluation and observation, the pre-set aim and objectives on the improvement of mental wellbeing in children and adolescents with SEN can be confirmed.

**Trial registration:**

ClinicalTrial.gov, NCT06112483. Registered on 31 October 2023.

**Supplementary Information:**

The online version contains supplementary material available at 10.1186/s12887-024-05288-8.

## Background

According to the Article 31 of the UN Convention on the Rights of the Child in 1989 states that “Every child has the right to rest and leisure, to engage in play and recreational activities appropriate to the age of the child and to participate freely in cultural life and the arts” [1]. Children’s play activities can take two different forms: structured play and unstructured play. Both are vital for a child’s wellbeing, learning, development and growth. Structured play, also known as goal-oriented play, generally involves using logic to solve problems, while unstructured play, or free play, is creative and open-ended. Unstructured play is sometimes called free play as it is creative and improvised with no set goal and unlimited possibilities. In Hong Kong, most of the children participate in structured play especially children with special educational needs (SEN) in the current situation in Hong Kong. This may be due to a lack of understanding of the benefits of unstructured play and how it can facilitate the growth and development of children, including promoting their mental health and wellbeing.

Special educational needs (SEN) refer to the learning needs of students arising from various types of physical and mental illnesses or conditions. According to Education Bureau of Hong Kong Government, there are nine types of special educational needs (SEN) including intellectual disability, autism spectrum disorder, attention deficit hyperactivity disorder, mental illness, specific learning difficulties, physical disability, visual impairment, hearing impairment and speech and language impairment [2]. In this study, we recruited SEN students with either mild or moderate intellectual and physical disabilities diagnosed with one or more types of special educational needs as shown in the above list [2].

### Cognitive learning theory

Bandura’s social learning theory has been adopted to observe the study participants’ behavioural change [3]. According to Bandura, people learn new behaviours by observing and imitating others. This theory also considers how both environmental and cognitive factors interact to influence human learning and behavioural change [3].

### Impact of COVID-19 on SEN children’s and adolescents’ mental welling

Children of today’s world are growing up in a dynamic environment in which they may not have enough time and opportunities to play freely. Children in Hong Kong are not getting enough outdoor free playtime, especially during the COVID-19 pandemic. Service disruption, school closure, social isolation, reduced outdoor activities and prolonged use of digital devices are associated with behavioural and emotional deterioration in SEN children in the context of COVID-19 pandemic [4]. The COVID-19 pandemic has had a serious impact on the mental health of populations. About 30% of adults and 34% of young people reported that their mental health worsened over the course of the pandemic [5]. As more young people return to the classroom, it is more important than ever for schools, colleges and universities to provide support to address the mental health concerns of students.

### The power of play and mental wellbeing

Unstructured play, or free play, is when a child can play at their leisure and explore in an unstructured way, with toys, resources, or materials available to use or do whatever they choose safely, for example, playing in a sandbox. It has been found that outdoor unstructured play-based interventions demonstrated a beneficial effect on positive mental health in children and adolescents with SEN. This makes a valuable contribution in presenting the state of evidence or lack thereof addressing the mental health needs of children with SEN. Unstructured play-based interventions appear to have a beneficial effect on promoting positive mental health in SEN children [6].

In Hong Kong, it is claimed that children’s playtime is significantly reduced or taken away by their schoolteachers and parents who ‘stole’ their spare playtime to invest more in schoolwork. Most schoolteachers and parents urged that play or sport and physical activity may hinder academic performance as students in Hong Kong are too busy to achieve academic success.

For those students are allowed to play, but they are restricted to play indoor with rules and regulations. There is no creativity, problem-solving skills, and minimum social engagement with others. Thus, children in Hong Kong may not be getting enough quality of playtime and space to facilitate their growth and development especially in promoting their mental-emotional wellbeing during their developmental stages.

A playful approach of unstructured play activities has also been found effective on promoting positive emotions and emotional competence among early adolescents [7]. Unstructured outdoor play coupled with mindfulness-based interventions via the 'Supporting Wellness in E-Child Learning Enviornment' (SWELE) program can foster the mental health and wellbeing of children and adolescents with SEN [8].

The gap of services is that the extra-curricular unstructured outdoor play with mindfulness-based interventions have not been included in the existing extra-curricular to address the mental health needs of SEN students (children and adolescents) returning to school, in the context of COVID-19 pandemic.

### Objectives

The aim of this study is to examine the effects of a SWELE program by implementing a play-based driver on supporting wellness in E-child learning environments by combining unstructured outdoor play activities with mindfulness-based interventions to promote mental wellbeing among SEN students, in the context of the COVID-19 pandemic.

Following participation in the SWELE program: SEN students’:

1. mood level will be increased.

2. anxiety symptoms (negative emotions) will be reduced.

3. self-regulations and social skills in peer interaction relationships will be improved; and

4. playfulness behaviors will be increased.

### Trial design

A convergent parallel mixed-methods design [9] both with a quantitative measure using a single group pre-test and post-test quasi-experiment behavioural observational method to examine the effects of a 16-week SWELE program [8] on SEN students’ mental welling; and with a qualitative design to conduct 16 focus group interviews involving schoolteachers, school principals, parents, SEN students, school nurses [10] in six special groups to explore the their perceptions and experiences after participated the SWELE program. The date for ClinicalTrials.gov Protocol Registration NCT06112483 was on 31 October 2023.

## Methods

SWELE program is a 16-week program combining unstructured outdoor play with mindfulness-based activities to promote mental wellbeing among SEN students, in the context of the COVID-19 pandemic. The protocol was prepared according to the SPIRIT checklist (Additional file 1).

This is a mixed methods study design. Study measurements will be conducted in the two-time points [baseline Pre-test and Post-intervention] for data collection using six questionnaires to evaluate the project objectives and impact in the following. Sixteen focus group interviews will also be conducted from six special schools after participated the SWELE program. The focus group interview may last 40–60 minutes.

There are six questionnaires in this study and their details are in the followings:

1. Demographic information sheet

The demographic sheet will include name, age, gender, grade and medical diagnosis. (see Appendix I)

2. Emoji Mood Scale

To be inclusive and accurately capture each SEN student’s self-report on mood and emotion, it is useful to present visual stimuli in association with text. The Emoji Mood Scale includes five different emoji with a 5-points response scale of mood ranging from 1–5: 1-Very bad; 2-bad, 3-so-so, 4-good; to 5-very good [11]. The range of scale score can be from 1 to 5 where higher scores represented very good mood and emotion. The mood scale with the Chinese descriptor for each emoji mood scale can be found in Appendix II.

3. Six-item Short-form of State-Trait Anxiety Inventory (STAI-6)

The version 6-item short-form of the STAI [12] is a sensitive to fluctuations in state anxiety. When compared with the full-form of the STAI, the six-item version offers a briefer and just as acceptable scale for subjects while maintaining results that are comparable to those obtained using the full-form of the STAI. The use of this six-item short-form (STAI-6) produced scores similar to those obtained using the full-form. Observers will be asked to answer each item using the following scale (Scale of 1–5; 1 = not at all / very slightly, 2 = a little, 3 = moderately, 4 = quite a bit, 5 = extremely). The range of score from 6 to 24. The highest score shows a high anxiety level of individual. Back translation of the STAI-6 had been performed after obtained the content validity index (CVI = 1.0). The Chinese version of STAI-6 has been attached in Appendix III. The short Chinese version of the STAI [13] demonstrates sound psychometric properties and is applicable in evaluating the level of anxiety in Chinese populations.

The development of a short Chinese version of the State-Trait Anxiety Inventory [13]. The aim of study was to develop and validate the short forms of the STAI scales, which are suitable for the situation in China, as studies have shown that the STAI is the most widely used tool for evaluating the state of anxiety.

Six-item versions of the STAI scales, which are used as brief screening tools, provide an opportunity to identify people who may benefit from treatment and thus improve the mental status of the general population. This scale expresses negative emotions and is interpreted as having negative directionality or lower scores imply less anxiety. Thus, early identification of anxiety is important for both the general and clinical populations. In particular, the influence of COVID-19 continues to interrupt regular mental health service delivery and it is not possible to accurately predict outbreaks and if people’s daily travels may be restricted. This short-form survey may help both clinicians and people who want to quickly evaluate their mood status in the clinic or at home.

4. Child Behaviour Rating Scale (CBRS)

Child Behaviour Rating Scale (CBRS) [14] is a teacher-report comprised of 17-items – 10 items assesses self-regulation and 7 items assesses social skills. The Classroom Self-Regulation subscale is comprised of 10 items that assess teachers’ perceptions of children’s behavioural regulation during academic tasks; the Social Skills subscale is comprised of 7 items that assess teachers’ perceptions of children’s behavioural regulation in social situations. This factor structure has been validated in other, and many who use the CBRS opt to utilize the items from the Classroom Self-Regulation subscale independently or in conjunction with those from the Social Skills subscale to assess children’s behavioural regulation

5. Children’s Playfulness Scale (CPS)

The Children’s Playfulness Scale (CPS) is used to assess children’s playfulness based on five dimensions of play among SEN students [15]. Lieberman proposed five manifestations of playfulness for the CPS: physical spontaneity, social spontaneity, cognitive spontaneity, manifest joy, and sense of humour.

Barnett developed a 23-item questionnaire based on these five dimensions of play [16]. Each item poses a statement such as “the child uses unconventional objects in play,” which is scored on a 5-point Likert-type scale with responses ranging from “sounds exactly like the child” to “doesn’t sound at all like the child” [16]. The modified version of this questionnaire for parents read “my child” rather than “the child” for each response. The measurement of playfulness of CPS was accomplished by posing statements and asking the rater to respond to each statement by choosing one of five response alternatives. Each item poses a statement such as “the child uses unconventional objects in play,” which is scored on a 5-point Likert-type scale with responses ranging from “sounds exactly like the child” to “doesn’t sound at all like the child” [16]. The modified version of this questionnaire for parents read “my child” rather than “the child” for each response. The CPS instrument has been shown to be reliable and valid with the inter- rater reliability among the trained teachers was found to exceed 0.87 [16].

### Population

Grade 1- Grade 12 SEN students (children and adolescents) will be enrolled from six selected special schools in Hong Kong. Inclusion criteria of study participants are: 1) students are studying in a special school; 2) students can speak and understand Cantonese; 3) students have no diagnosis of any cardiovascular disease; 4) parental consent obtained. Exclusion criteria of the study are: 1) students are not studying in the special schools; 2) students do not speak and understand Cantonese or English; 3) student has diagnosis of cardiovascular disease; 4) student cannot provide parental consent.

**Fig. 1:**
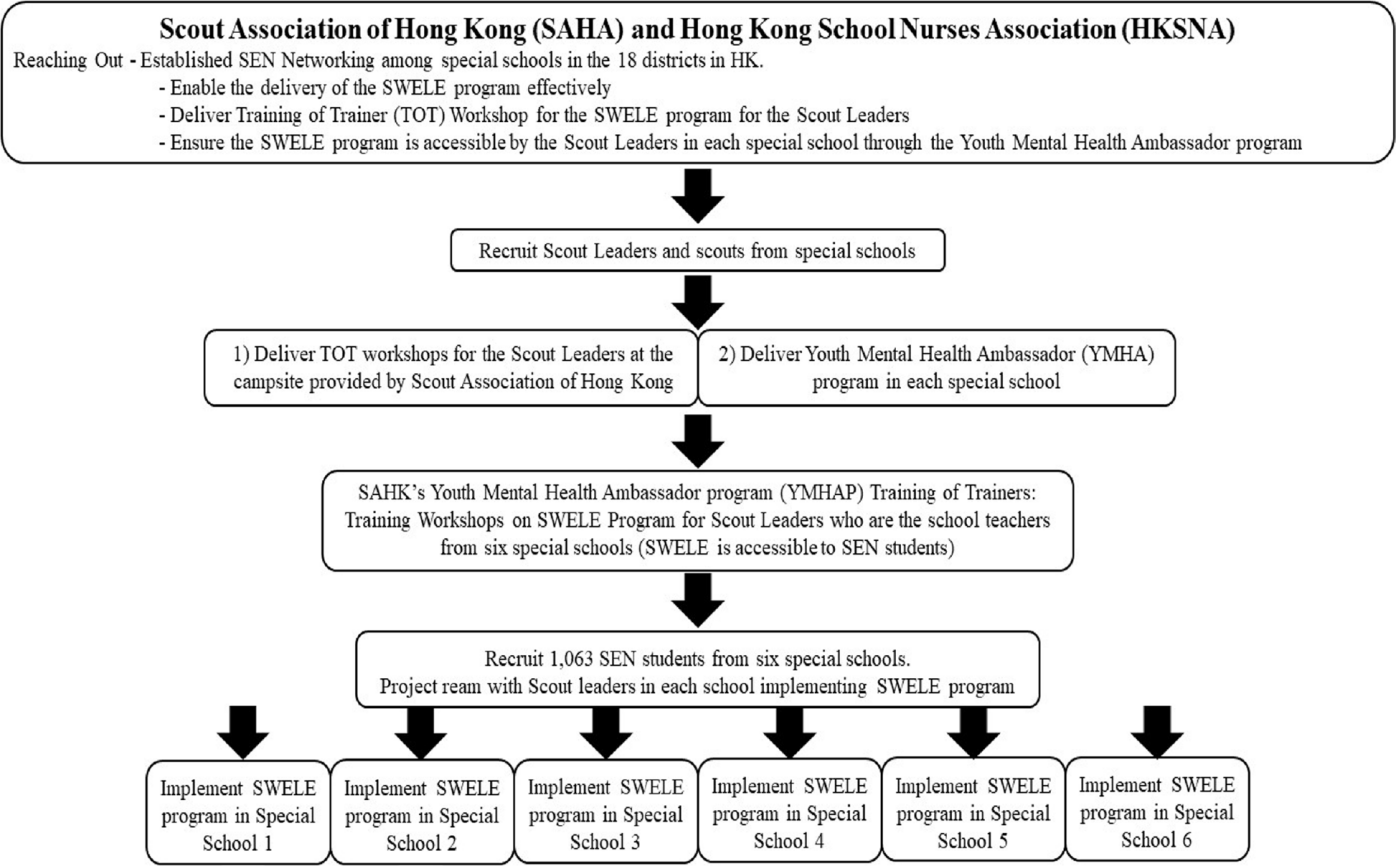
“SWELE program” Reaching Out and accessible to six special schools in Hong Kong

There will be 1,063 children and adolescents aged 6–18 years old with mild to moderate physical and intellectual disabilities (ID) from the six selected special schools will be recruited to participate the SWELE program implemented by the research team (Fig. 1). Based on our team’s experience, it is anticipated that at least 90% of them are eligible and willing to join the program, and the attrition rate would be less than 10%. It is therefore expected to have at least 850 children completing the program and outcome assessments. Such a sample size (*n* = 850) allows to detect a change in an outcome of effect size as small as 0.1 with 80% power at a level of significance of 0.05 (2-sided). An effect size of 0.1 is conventionally regarded as small [17].

### Intervention: SWELE program

The SWELE program combine unstructured outdoor play with mindfulness-based interventions is therefore different from other existing services in the community. This 16-week SWELE program focuses on promoting mental wellbeing by combining unstructured outdoor play with mindfulness-based interventions. The details of the SWELE program can be found in Table 2. There is no such SWELE program to promote mental wellbeing for children and adolescents with SEN in Hong Kong, especially in the context of COVID-19 pandemic.

A playful approach of unstructured play has also been found effective on promoting positive emotions and emotional competence among early adolescents. Unstructured outdoor play coupled with mindfulness-based interventions via SWELE Program to Promote Mental Health for children and adolescents with SEN during COVID-19 pandemic. There is limited unstructured outdoor play-based program integrated into the extra-curricular activities in the special schools in Hong Kong.

The COVID-19 pandemic has resulted in increased anxiety and stress among children and adolescents in Hong Kong. To help alleviate these symptoms, the project team of the SWELE program is collaborating with The Scout Association of Hong Kong on a 16-week SWELE program to promote SEN students’ mental wellbeing. The mindfulness-based interventions focus on social-emotional learning activities in schools. Without intentional social-emotional development, SEN students may not learn how to process their emotions and connect with others in healthy ways. That is where mindfulness can come in. Mindfulness involves both an awareness and an acceptance of both the world around us and our internal experiences. Mindful people tend to focus more on the present instead of reflecting on the past or future, and they cultivate a curiosity towards their thoughts, emotions, or physical sensations.

The new knowledge generates from the findings of this mental health promotion project are important for raising the public’s awareness about the impact on SEN student’s mental wellbeing and advancing our understanding of the COVID-19 pandemic’s impacts on service disruption and transition and subsequently on concerning SEN students’ mental health. Thus, school health policy and strategies could be developed appropriately to promote the mental wellbeing of children and adolescents with SEN.

Six special schools have been recruited and will participate in the SWELE program. This is a 16-week SWELE program, and it will be conducted in two batches. Each batch has three special schools. A SWELE program play schedule with the different age groups/classes prepared by the schoolteachers will be given to the SWELE team so that they can plan the age-appropriate activities on those scheduled dates. Table 1 demonstrate the key milestones in implementing and evaluating the SWELE program. Figure 1 illustrates a flow chart with steps for the recruitment of SEN students. Table 2 lists the key activities of the SWELE program.

**Table 1:**
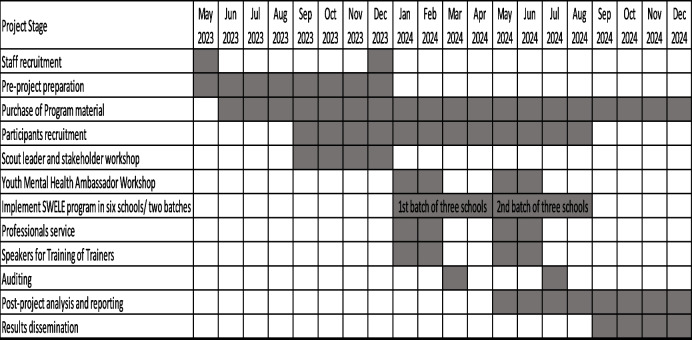
Project Milestones of a SWELE Program

**Table 2:** A 16-week SWELE program – extracurricular activities to promote SEN children’s and adolescents’ mental wellbeing status

SWELE Activities for age-appropriate groups /classes	Frequency/Schedule	Venue
(Training of Trainers) Training Workshop for Scout Leaders, School Nurses and Schoolteacher	One Whole Day	School Hall/Classroom
Training Workshop for Youth Mental Health Ambassador Program (SEN Students)	One Whole Day	School Hall/Classroom
Seminar for parents, school social worker, school nurses	Half a Day	School Hall/Classroom
Outdoor playground age-appropriate activities / games (using natural and recycle materials: branches, tyres, sand, water, leaves, boxes, plastic bottles, tools/toys…) Tunnel-through-boxes, Tyres Sand and water play, Tree House & Adventure Course Wobbly Wood Oasis Hut Merchant Play Ship with Pirate Tower. Play Ship Nina Sky climber with Artwork	35 min each time and 2 times per week for 16 weeks	School playground
Indoor mindfulness interventions	10 min each time and 2 times per week for 16 weeks	School Hall/Classroom: Storytelling, soft music, video, mindful arts
Mental Health Campaign	Once a month for four times	Games, smiling stickers, posters, emoji
‘Grow Your Mind’ Award with Certificate	One time only	Identify strengths on students and teacher-focus on connect, succeed, thrive and how this enable them to be productive and life-long learners

### Main activities of SWELE program:

- Unstructured outdoor free play integrated into the school’s extra-curricular activities (Youth Mental Health Ambassadors) to promote SEN students’ mental health for objectives 1–4.
- Use mindfulness-based podcasts, mindfulness games, mindfulness art for objectives 1–2.
- Through meditation and deep breathing technique, storytelling with relaxing waves piano music, yoga and mindful art for objectives 1–2.
- Training Workshops (Training of trainers) for Scout Leaders who will implement SWELE program in each special school.
- Youth Mental Health Ambassador Program for SEN students who are enrolled in Scout Club in each special school; SWELE training workshops for parents and schoolteachers will also be held in each special school for objectives 1–4.
- Examples of unstructured play might be creative play alone or with others, including artistic or musical games. imaginative games – for example, making cubbyhouses with boxes or blankets, dressing up or playing make-believe, exploring new or favorite spaces like school backyards, parks, playgrounds and so on.

The key milestones to implement the SWELE program can be found in Table 1. The SWELE program is a 16-week program and the main activities include: One training workshop for three groups in each special school: 1) Scout Leaders (special schools’ teachers), 2) SEN students (scouts in the school) to become Youth Mental Health Ambassadors and 3) for parents, school social workers, school nurses, schoolteachers and stakeholders; 45 min unstructured outdoor play with mindfulness activities two times per week for 16 weeks (see Table 1 below).

### Ethical considerations

Ethical approval was obtained from the Joint Chinese University of Hong Kong and New Territories East Cluster Clinical Research Ethics Committee prior to conducting the study (CREC Ref. No: 2022.659-T) registered on 23 March 2023. The study complies with the declaration of Helsinki with a statement of ethical principles for medical research involving humans. The ICH-GCP E6(R3) standards which is a harmonised standard that protects the rights, safety and welfare of human subjects and minimises their exposure to risk products [18]. Parental consents will be obtained through the School Communication System. Information sheet and parental consents will be distributed to parents through the school communication mails. Each participant’s questionnaire will be coded with a number. School and students’ names will not be released in the publications. Students can withdraw from the study anytime without any penalty in their routines and schooling. All the research data will be kept in a locked cabinet to protect the participant’s privacy.

### Outcome measures in the SWELE program

#### Primary outcomes

After participated the SWELE program, the primary outcomes include reducing anxiety symptoms, reducing negative emotions, improving social skills in peer relationships; and changing in playfulness level among SEN children and adolescents.

For objective (1), to increase over 80% of SEN students’ happy mood after participated the SWELE program.

For objective (2), to reduce over 80% of SEN students’ anxiety symptoms (negative emotions) after participated the SWELE program.

For objective (3), to improve over 80% of SEN students’ social skills in peer relationships after participated the SWELE program; and.

For objective (4), to improve over 70% of the SEN students’ playfulness level after participated the SWELE program.

### Data collection

#### Procedure for data collection for quantitative method

Prior to commencing the study, a briefing session will be delivered to the direct care staff in the school, school nurses and schoolteachers who will assist the participants to complete the questionnaire and demographic sheet with their understanding and behavioural observations.

The study will be conducted with the support of and in partnership with the special schools’ school principals, schoolteachers, school nurses and school social workers as they all play a critical role in supporting the social and emotional development of SEN children and adolescents in the special schools. This is a before and after intervention data collection for quasi-experimental and observational study.

Ethical approval form and parental consents will be obtained prior to implementing the SWELE program in the six selected special schools.

Pre-test data collection will be conducted prior to starting the intervention (a 16-week SWELE program) for target participants. The research team members, student helpers and school nurses will work with schoolteachers to implement 16 sessions (45–60 min per session) for 16 weeks. The details of the activities of this intervention as shown in Table 1 above. Post-test data collection will be conducted immediately after participated the SWELE -program.

#### Procedure for data collection for the focus Group Interviews

There will be sixteen focus group interviews with 8–10 participants including schoolteachers, school principals, school nurses, SEN students and their parents will be conducted to gain an in-depth understanding of the views and experiences after participated in the SWELE program and to explore the mental wellbeing of the SEN students participating in the SWELE program. Interview guide will be developed based on literature review as follows:

#### Interview guides:

1. Tell me the experiences in participating the SWELE program.
2. What do you like the best of the SWELE program?
3. What do you like the least of the SWELE program?
4. Would you recommend the SWELE program to your friends and why?
5. Anything you would like the SWELE program to be improved?

### Statistical analysis

Appropriate descriptive statistics, such as mean (standard deviation), median (inter-quartile range) and frequency (percentage) will be used to summarize and present the baseline characteristics and outcome data of the participants. Normality of continuous variables will be assessed based on their skewness and kurtosis statistics, values within ± 2 indicating the plausibility of normal distribution [19]. Suitable transformations will be made on skewed variables before subjecting them into inferential analysis. Paired sample t-test will be used to compare the change of each outcome (mood, anxiety, social, playfulness) at post-test with respect to pre-test within group and subgroups (age and gender). All statistical analyses will be performed using IBM SPSS 28 (IBM Crop., Armonk, NY) with level of significance set at 0.05.

## Discussion

SWELE Program allows SEN students to explore the environment in a creative way to learn more about themselves. This indirectly promotes the mental wellbeing of SEN students through unstructured play activities and mindfulness-based interventions. Overscheduling structured play often leads to a child developing stress and anxiety, as well as the possibility of depression.

A quasi-experimental design will be used to evaluate the impact of the SWELE intervention on the four emotional and social aspects of SEN students before and after the intervention because of two major reasons:

1. It is not logistically feasible to (i) randomise students in a school since there is not enough manpower to monitor or care for students in different groups. (ii) to use school as the randomisation unit as the schools cannot offer flexible schedules for our activities.
2. There are ethical concerns about withholding intervention from a control group in this vulnerable population.

While we acknowledge the quasi-experimental design limits the ability to rule out a placebo effect, the study findings will explore the potential influence of such impact in the qualitative study.

A child’s mental wellbeing may be affected by different levels of play activities. Children who have more unstructured outdoor play experience higher levels of self-reflection and creative growth. In turn, these abilities help lower their levels of stress and anxiety. Thus, unstructured play helps children process difficulty emotions more directly and effectively.

A key implication of this SWELE program is that the findings can add the value of unstructured outdoor play to raise public awareness among SEN educators, practitioners, stakeholders (parents, schoolteachers, school principals) to understand the impact of COVID-19 pandemic on the mental wellbeing of SEN students and the benefits of implementing an unstructured play-based intervention with mindfulness activities can promote SEN students’ mental-emotional wellbeing. It is an imperative to better understand the benefits and possible drawbacks of using unstructured play activities coupled with mindfulness-based interventions to support the mental health of SEN children and adolescents with social, communication, and or language impairments so SEN educators and stakeholders can make informed choices about treatment for SEN children and adolescents.

Giving children space to play freely outdoor allows them to work through feelings such as pain, fear or loss while being able to still act like a child. Play gives them a way to express things they are struggling with and may not have the words to fully explain. Unstructured outdoor play can facilitate children to recognise and express their emotions and develop positive relationships with peers and family members. It also helps them to deal with anxiety and boredom and build their ability to concentrate and focus on what is important to them. It is anticipated that the impact of the SWELE program will evidence the need to prioritise interventions investigating the efficacy of unstructured play-based interventions to support the mental health of children and adolescents with SEN.

It is anticipated that the SWELE program will bring significant added value to the existing program being carried out by the schools. Many do not fully understand the concept of unstructured free play and the benefits it offers to children, especially in promoting children’s mental wellbeing. A UNICEF study reported that about 84% of parents misunderstood what free play meant [20]. Children and adolescents need the space to set the rules and direction of their learning for the activity to count as free play. Unstructured outdoor play can facilitate children to recognise and express their emotions and develop positive relationships with peers and family members. It also helps them to deal with anxiety and boredom and build their ability to concentrate and focus on what's important to them. Thus, the needs for promoting their emotional and wellbeing can be addressed through unstructured free play with mindfulness-based interventions [8, 10].

## Conclusions

Recognising the impact of COVID-19 pandemic on SEN students’ mental health is necessary as the pandemic has impacted upon the mental wellbeing of SEN children and adolescents and may have contributed to their academic and behavioural problems. Post-traumatic stress disorder (PTSD) is one of the most common negative psychological reactions with emotional and behavioural problems, and academic boredom with school closures is a typical academic issue arising from the pandemic. PTSD might be related to academic boredom, services disruption, social isolation, reduced outdoor activities, school closures, reduced outdoor activities and increased digital time during the COVID-19 pandemic. Therefore, the implementation of the SWELE program can be one of the strategies to reduce unforeseen impact on the mental health and wellbeing of SEN children and adolescents. This type of extra-curricular unstructured outdoor play with mindfulness-based interventions have not been included in the existing extra-curricular to address the mental health needs of SEN students return to school, in the context of COVID-19 pandemic.

There is limited mental health services to address the social-emotional needs of children and adolescents with SEN when they returned to schools after the long school closures due to COVID-19 pandemic in Hong Kong. The core activities of the SWELE program focus on the development of two core social-emotional skills: self-regulations and self-awareness via mindfulness-based interventions.

## Supplementary Information

Supplementary Material 1.

Supplementary Material 2.

Supplementary Material 3.

Supplementary Material 4.

Supplementary Material 5.

Supplementary Material 6.

## Data Availability

No datasets were generated or analysed during the current study.

## Abbreviations

CBRS: Child behaviour rating scale
COVID-19: Coronavirus disease 2019
CPS: Children’s playfulness scale
PTSD: Post-traumatic stress disorder
TOT: Training of trainers
SEN: Special educational needs
STAI: State-trait anxiety inventory
SWELE: Supporting wellness in E-child learning environments
