# Supplementary material for: Effects of a SWELE program for improving mental wellbeing in children and adolescents with special educational needs: protocol of a quasi-experimental study
Source: BMC Pediatr. 2024 Dec 6;24:800. doi: 10.1186/s12887-024-05288-8 (PMC11622570; doi:10.1186/s12887-024-05288-8)
Supplement: Supplementary file 1 — Supplementary Material 2. [file 12887_2024_5288_MOESM1_ESM.docx]

**Appendix I – Demographic Information Sheet**

**(A) Demographic information**

Name: __________________________

| Gender: | | | □ Male | | | □ Female | | | | | | | | | |  |  |  |
| --- | --- | --- | --- | --- | --- | --- | --- | --- | --- | --- | --- | --- | --- | --- | --- | --- | --- | --- |
| Age: | □ 6 | | | □ 7 | | | □ 8 | | □ 9 | | □ 10 | | □ 11 | □ 12 | | | □ 13 | |
|  | □ 14 | | | □ 15 | | | □ 16 | | □ 17 | | □ 18 | | □ 19 | □ 20 or above | | | | |
| Grade: | | □ P1 | | | □ P2 | | | □ P3 | | □ P4 | | □ P5 | | | □ P6 | | |  |
|  | | □ S1 | | | □ S2 | | | □ S3 | | □ S4 | | □ S5 | | | □ S6 | | |  |
