# Supplementary figures and images for: Effects of a SWELE program for improving mental wellbeing in children and adolescents with special educational needs: protocol of a quasi-experimental study
Source: BMC Pediatr. 2024 Dec 6;24:800. doi: 10.1186/s12887-024-05288-8 (PMC11622570; doi:10.1186/s12887-024-05288-8)

**Appendix II – Mood Scale**

(B) Emoji Mood Scale


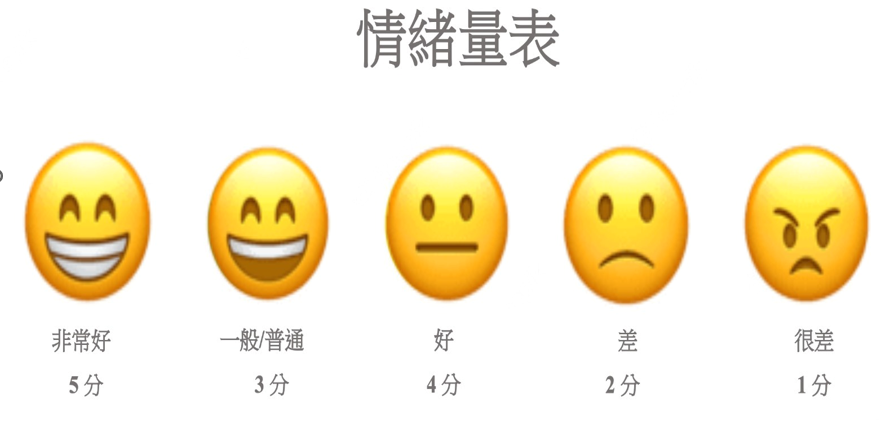


|  |
| --- |

Supplement: Supplementary file 2 — Supplementary Material 3. [file 12887_2024_5288_MOESM2_ESM.docx]
