# Supplementary material for: Effects of a SWELE program for improving mental wellbeing in children and adolescents with special educational needs: protocol of a quasi-experimental study
Source: BMC Pediatr. 2024 Dec 6;24:800. doi: 10.1186/s12887-024-05288-8 (PMC11622570; doi:10.1186/s12887-024-05288-8)
Supplement: Supplementary file 3 — Supplementary Material 4. [file 12887_2024_5288_MOESM3_ESM.docx]

**Appendix III – Short version of Stat-Trait Anxiety Inventory (STAI)**

(C) STAI

| Domains / Items | Not at all | Somewhat | Moderately so | Very much so |
| --- | --- | --- | --- | --- |
| Here’s how you feel at the moment. |  |  |  |  |
| 1. I feel at ease. | 1 | 2 | 3 | 4 |
| 2. I feel comfortable. | 1 | 2 | 3 | 4 |
| 3. I am relaxed. | 1 | 2 | 3 | 4 |
| 4. I feel content. | 1 | 2 | 3 | 4 |
| 5. I feel steady. | 1 | 2 | 3 | 4 |
| 6. I feel pleasant. | 1 | 2 | 3 | 4 |
| Here’s how you feel often. |  |  |  |  |
| 1. I feel pleasant. | 1 | 2 | 3 | 4 |
| 2. I feel satisfied with myself. | 1 | 2 | 3 | 4 |
| 3. I feel rested. | 1 | 2 | 3 | 4 |
| 4. I am calm, cool and collected. | 1 | 2 | 3 | 4 |
| 5. I am happy. | 1 | 2 | 3 | 4 |
| 6. I feel secure. | 1 | 2 | 3 | 4 |
| 7. I am content. | 1 | 2 | 3 | 4 |

|  |
| --- |
