# Supplementary material for: Effects of a SWELE program for improving mental wellbeing in children and adolescents with special educational needs: protocol of a quasi-experimental study
Source: BMC Pediatr. 2024 Dec 6;24:800. doi: 10.1186/s12887-024-05288-8 (PMC11622570; doi:10.1186/s12887-024-05288-8)
Supplement: Supplementary file 4 — Supplementary Material 5. [file 12887_2024_5288_MOESM4_ESM.docx]

**Appendix IV – Self-regulation and Social Skills Scale**

(D) Self-regulation and social skills

| Items | Never | Seldom | Sometimes | Often | Always |
| --- | --- | --- | --- | --- | --- |
| **Classroom Self-regulation** |  |  |  |  |  |
| 1. Observes rules and follows direction without reminders. | 1 | 2 | 3 | 4 | 5 |
| 2. Completes learning tasks in an organized way. | 1 | 2 | 3 | 4 | 5 |
| 3. Complete tasks successfully. | 1 | 2 | 3 | 4 | 5 |
| 4. Attempts new and challenging tasks. | 1 | 2 | 3 | 4 | 5 |
| 5. Concentrate when working, not easily distracted. | 1 | 2 | 3 | 4 | 5 |
| 6. Responds to instructions and begins appropriate task. | 1 | 2 | 3 | 4 | 5 |
| 7. Takes time to do his/her best work. | 1 | 2 | 3 | 4 | 5 |
| 8. Finds and organizes materials. | 1 | 2 | 3 | 4 | 5 |
| 9. See own errors on tasks and correct them. | 1 | 2 | 3 | 4 | 5 |
| 10. Returns to unfinished tasks after interruption. | 1 | 2 | 3 | 4 | 5 |
| **Social Skills (Interpersonal Skills)** |  |  |  |  |  |
| 1. Willing to share. | 1 | 2 | 3 | 4 | 5 |
| 2. Expresses hostility – Verbally. | 1 | 2 | 3 | 4 | 5 |
| 3. Expresses hostility – Physically. | 1 | 2 | 3 | 4 | 5 |
| 4. Cooperate with playmates. | 1 | 2 | 3 | 4 | 5 |
| 5. Take turns without being told to do so. | 1 | 2 | 3 | 4 | 5 |
| 6. Complies with adult directives – With little or no resistance. | 1 | 2 | 3 | 4 | 5 |
| 7. Does not fuss when doesn’t get teacher’s attention. | 1 | 2 | 3 | 4 | 5 |
