# Supplementary material for: Effects of a SWELE program for improving mental wellbeing in children and adolescents with special educational needs: protocol of a quasi-experimental study
Source: BMC Pediatr. 2024 Dec 6;24:800. doi: 10.1186/s12887-024-05288-8 (PMC11622570; doi:10.1186/s12887-024-05288-8)
Supplement: Supplementary file 5 — Supplementary Material 6. [file 12887_2024_5288_MOESM5_ESM.docx]

**Appendix V – Children’s Playfulness Scale (CPS-23)**

(E) Children’s playfulness behaviors

| Items | Almost never true for me | Seldom true for me | Sometimes true for me | Often true for me | Almost always true for me |
| --- | --- | --- | --- | --- | --- |
| **I. Social Spontaneity** |  |  |  |  |  |
| 1. The child assumes a leadership role when playing with others. | 1 | 2 | 3 | 4 | 5 |
| 2. The child initiates play with others. | 1 | 2 | 3 | 4 | 5 |
| 3. The child is willing to share playthings | 1 | 2 | 3 | 4 | 5 |
| 4. The child responds easily to others’ approaches during play. | 1 | 2 | 3 | 4 | 5 |
| 5. The child plays cooperatively with other children. | 1 | 2 | 3 | 4 | 5 |
| **II. Cognitive spontaneity** |  |  |  |  |  |
| 6. The child invent his/her own game to play. | 1 | 2 | 3 | 4 | 5 |
| 7. The Child stays with one activity rather than changes activity during play. | 1 | 2 | 3 | 4 | 5 |
| 8. The child assumes different character roles in play. | 1 | 2 | 3 | 4 | 5 |
| 9. The child uses unconventional objects in play | 1 | 2 | 3 | 4 | 5 |
| **III. Physical spontaneity** |  |  |  |  |  |
| 10. The child runs (skips, hops, jumps) a lot in play. | 1 | 2 | 3 | 4 | 5 |
| 11. The child prefers to be active rather than quiet in play. | 1 | 2 | 3 | 4 | 5 |
| 12. The child is physically active during play. | 1 | 2 | 3 | 4 | 5 |
| 13. The child’s movement are generally well-coordinated during play activities. | 1 | 2 | 3 | 4 | 5 |
| **IV. Sense of humor** |  |  |  |  |  |
| 14. The child enjoys joking with other children. | 1 | 2 | 3 | 4 | 5 |
| 15. The child tells funny stories | 1 | 2 | 3 | 4 | 5 |
| 16. The child gently teases others while at play. | 1 | 2 | 3 | 4 | 5 |
| 17. The child laughs at funny stories. | 1 | 2 | 3 | 4 | 5 |
| 18. The child likes to clown around in play. | 1 | 2 | 3 | 4 | 5 |
| **V. Manifest Joy** |  |  |  |  |  |
| 19. The child sings and talks while playing. | 1 | 2 | 3 | 4 | 5 |
| 20. The child shows enthusiasm during play. | 1 | 2 | 3 | 4 | 5 |
| 21. The child expresses enjoyment during play. | 1 | 2 | 3 | 4 | 5 |
| 22. The child demonstrates exuberance during play. | 1 | 2 | 3 | 4 | 5 |
| 23. The child is restrained in expressing emotion during play. | 1 | 2 | 3 | 4 | 5 |
